
JOURNAL INFORMATION
==============================
NLM Title Abbreviation: ScientificWorldJournal
Journal ID: ScientificWorldJournal
NLM Journal ID: 101131163
Journal ID: TSWJ

The Scientific World Journal

ISSN: 2356-6140
EISSN: 1537-744X
Publisher: Wiley

ARTICLE INFORMATION
==============================
PMCID: PMC5763672
PMID: 20602080
DOI: 10.1100/tsw.2010.119
Article ID: 345197
Article version: 1
Subjects: Directions in Science

Mesenchymal Stem Cells: “Repair Cells” that Serve Wounds and Cancer?

Dittmer Jürgen *
Klinik für Gynäkologie, Universität Halle, Halle (Saale), Germany
*Jürgen Dittmer: juergen.dittmer@medizin.uni-halle.deAcademic Editor: Graham V Lees

Publication date: 2010
Electronic publication date: 2010 Jun 29
Volume: 10
First page: 1234
Last page: 1238
Received 2010 Feb 18; Accepted 2010 May 18
Copyright: Copyright © 2010 Jürgen Dittmer.
Copyright year: 2010
License: This is an open access article distributed under the Creative Commons Attribution License, which permits unrestricted use, distribution, and reproduction in any medium, provided the original work is properly cited.
License URL: https://creativecommons.org/licenses/by/3.0/

Keywords: MSC, E-cadherin, ADAM10, breast cancer, migration

==============================
Directions in Science
*TheScientificWorldJOURNAL* (2010) 10, 1234–1238
ISSN 1537-744X; DOI 10.1100/tsw.2010.119

TheScientificWorldJOURNAL
www.thescientificworld.com

### Mesenchymal Stem Cells: "Repair Cells" that Serve Wounds and Cancer?

Jürgen Dittmer
*Klinik für Gynäkologie, Universität Halle, Halle (Saale), Germany*

**E-mail**: juergen.dittmer@medizin.uni-halle.de

*Received February 18, 2010; Accepted May 18, 2010; Published June 29, 2010*

**KEYWORDS**: MSC, E-cadherin, ADAM10, breast cancer, migration

The interaction between epithelial and stroma-forming mesenchymal cells, such as fibroblasts, plays a fundamental role in the development of both organs and tumors[1]. This cross-talk is bidirectional and usually paracrine in nature, which means that cells of the stromal compartment produce certain growth factors, cytokines, or chemokines, which act on neighboring epithelial cells and *vice versa*. Different types of mesenchymal cells are involved in this communication. Recently, a "new" type of mesenchymal cell, the mesenchymal stem cell (MSC) or multipotent mesenchymal stromal cell, has received much attention because it may play an important role in tissue regeneration and tumor progression[2]. The importance of the MSC, discovered in 1966 by Friedenstein[3], has only recently begun to be appreciated. MSCs are bone marrow–derived stromal cells with a fibroblast-like appearance that not only colonize numerous organs, but also are attracted to wounds and solid tumors especially. MSCs are defined by a number of features, which include their ability to differentiate into cells of mesodermal lineages, such as bone, cartilage, and fat cells[4]. In addition, MSCs may transdifferentiate into cells of ecto- or endodermal lineages, such as nerve, muscle, and epithelial cells[2]. It seems that, in wound healing, MSCs play the role of "repair cells" by entering the wound and differentiating to the kind of cells that are specifically needed to rebuild the tissue. The plasticity of these cells, combined with their migratory potential and their preference for injured tissue, makes MSCs an ideal tool for therapeutic tissue regeneration[5]. However, MSCs also enter tumors because they apparently "mistake" the tumors for normal wounds[6]. This response occurs because cancer cells secrete chemokines along with other proteins that attract MSCs, and increase their migratory activity[7,8]. In the tumor, MSCs may alter the behavior of the cancer cells. In addition, MSCs may differentiate to carcinoma-associated fibroblasts (CAF), which are known to be involved in cancer progression[9].

While a number of effects of MSCs on cancer cells has been described, little is known about the mechanisms involved[6]. Our recent work published in *Cellular and Molecular Life Sciences* suggests that, in breast cancer, human MSCs (hMSCs) target ADAM10 (a disintegrin and metalloproteinase 10)[10]. ADAM10 belongs to the ADAM family of transmembrane and secreted proteins, which include proteases that induce limited proteolysis of extracellular domains (ectodomains) of membrane-bound proteins, a process called shedding[11]. By cleaving the ectodomain of adhesion proteins, such as CD44, E-cadherin, or CX3 chemokine ligand 1, ADAM10 regulates cell adhesion and migration[12,13]. Overexpression of ADAM-10 has been reported in many tumors, suggesting that this protein may contribute to cancer progression[14,15,16].

©2010 with author.
Published by TheScientificWorld; www.thescientificworld.com

Previous studies undertaken in order to investigate the role of MSCs in tumor progression have primarily focused on the effect of MSCs on tumor growth. Most of these studies showed that MSCs stimulate cancer cell growth *in vitro* and *in vivo*[17,18,19,20]. Of note, in immunocompetent mice, this growth-stimulatory effect of MSCs on cancer may also be indirect, as MSCs are able to down-modulate the activities of various immune cells, including anticancer effector cells[2,20,21]. In addition to their growth-stimulatory activity, MSCs have also been shown to cause breast cancer cells to become more metastatic[19]. This effect seems to be mediated by the chemokine CCL5 (Rantes)[19], one of the many proteins secreted by MSCs[22]. CCL5 is recognized by the CCR5 receptor expressed on breast cancer cells. Even so, some studies suggest that MSCs may have antitumor activities as well[23,24,25,26].

There is also evidence that MSCs may alter cell-cell adhesion[27,28]. We addressed this occurrence by using 3-dimensional (3D) cultures of breast cancer cells. In 3D cultures, breast cancer cells form either spheroids or aggregates of irregular shape[29]. Spheroids are generated by less aggressive breast cancer cells, which still retain characteristics of normal breast epithelial cells. Also, spheroids are thought to recapitulate to some degree the glandular structure *in vivo*[30] and hence provide an *in vitro* experimental model more closely mimicking *in vivo* conditions. When we added GFP (green fluorescent protein)–labeled hMSCs to breast cancer spheroids, we found that the MSCs enter the spheroids and alter spheroid morphology. Immunohistochemical analysis revealed that cell-cell adhesion was disturbed in hMSC-treated spheroids. With the cell-cell adhesion weakened, breast cancer cells also showed higher motility. The main reason for these changes seemed to be the shedding of E-cadherin, the major glue between epithelial cells. In search for the mechanism, we observed that E-cadherin shedding and hMSC-induced migration could be blocked by both the ADAM10 inhibitor GI254023X and by an ADAM10-specific small-interference RNA. This outcome indicated that hMSC targeted ADAM10 to induce E-cadherin fragmentation, which then triggered the loss of cell-cell contact and cell migration (Fig. 1). Given these data, we wondered whether E-cadherin–deficient breast cancer cells would also benefit from hMSCs. Loss of E-cadherin is often observed in the course of cancer progression, resulting in cells with a mesenchymal phenotype characterized, among others, by fibroblast-like morphology, higher motility, and by a failure to form spheroids in 3D cultures[29,31]. When such E-cadherin–deficient breast cancer cells were incubated with hMSCs, migration was only marginally up-regulated, suggesting that E-cadherin/ADAM10 is indeed a major route through which hMSCs manipulate breast cancer cells. To stimulate ADAM10, either hMSCs could interact with breast cancer cells directly or they could secrete a soluble factor to which the cancer cells respond. To test this premise, we examined the effect of hMSC-conditioned medium, a cell-free medium that contains factors secreted by MSCs, on breast cancer migration. We learned that the conditioned medium was almost as effective as hMSCs in promoting breast cancer motility.

The acquisition of the migratory potential is an important step in cancer progression, allowing cancer cells to migrate into adjacent tissue[32]. Our data suggest that hMSCs enhance this potential by activating a protease that down-regulates cell-cell adhesion and, thereby, most likely promoting cancer progression. Interestingly, we found that hMSCs have little effect on the motility of more aggressive mesenchymal breast cancer cells that already had lost E-cadherin[10]. Instead, these *per se* highly motile cells "benefit" from the interaction with hMSCs in a different way in that they acquire an increased potential to metastasize[19]. It almost seems as though hMSCs modulate the behavior of breast cancer cells in the way the cancer benefits most. This result is consistent with the idea that the hMSC is a "repair cell", one that serves injured tissue by meeting their specific need for efficient healing. Cancer is regarded as "a wound that never heals". Hence it may be that by desperately trying to heal the "cancerous wound", hMSCs ironically promote cancer progression instead. Yet, currently too little is known about hMSCs to get a clearer picture of what the functions of hMSCs are in cancer progression. Among the many questions that remain are whether hMSCs act primarily on cancer cells as stem cells or as differentiated cells, such as CAFs, and whether, under certain conditions, hMSCs may actually heal "cancerous wounds", which would explain why, in some cases, hMSCs suppress cancer growth.

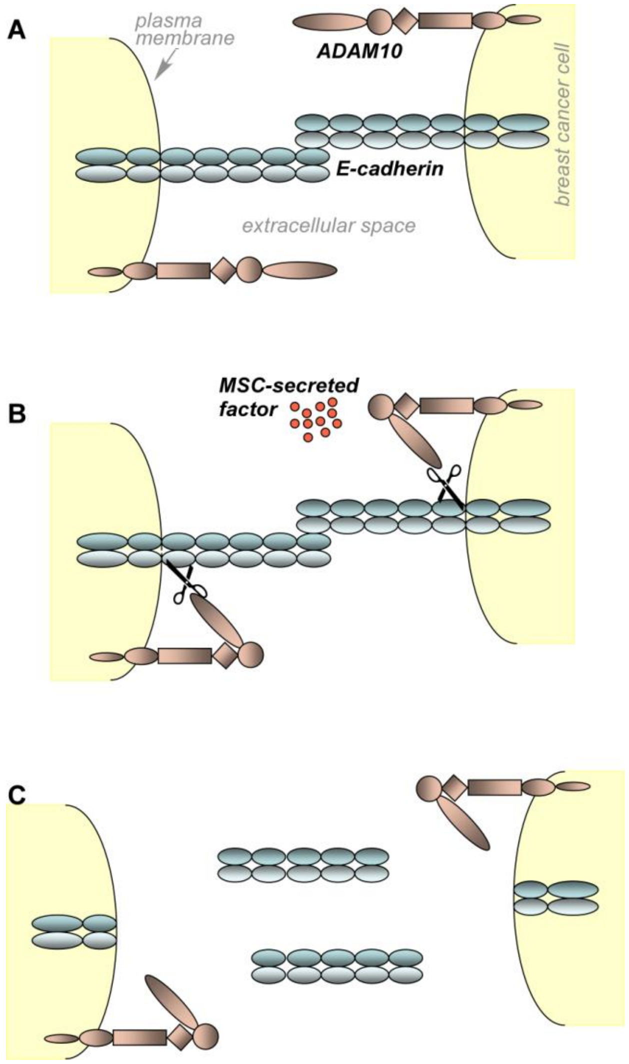

**FIGURE 1.** E-cadherin cell-cell adhesion of breast cancer cells (A) is weakened by a MSC-secreted factor that activates ADAM10 (B). Once detached from their neighboring cells, cells are able to migrate (C).

### ACKNOWLEDGMENT

I thank Dr. Charlotte Robidoux for critically reading the manuscript.

### REFERENCES

1. Nelson, C.M. and Bissell, M.J. (2006) Of extracellular matrix, scaffolds, and signaling: tissue architecture regulates development, homeostasis, and cancer. *Annu. Rev. Cell Dev. Biol.* **22**, 287–309.
2. Uccelli, A., Moretta, L., and Pistoia, V. (2008) Mesenchymal stem cells in health and disease. *Nat. Rev. Immunol.* **8**, 726–736.
3. Friedenstein, A.J., Piatetzky, S., II, and Petrakova, K.V. (1966) Osteogenesis in transplants of bone marrow cells. *J. Embryol. Exp. Morphol.* **16**, 381–390.
4. Dominici, M., Le Blanc, K., Mueller, I., Slaper-Cortenbach, I., Marini, F., Krause, D., Deans, R., Keating, A., Prockop, D., and Horwitz, E. (2006) Minimal criteria for defining multipotent mesenchymal stromal cells. The International Society for Cellular Therapy position statement. *Cytotherapy* **8**, 315–317.

5. Brooke, G., Cook, M., Blair, C., Han, R., Heazlewood, C., Jones, B., Kambouris, M., Kollar, K., McTaggart, S., Pelekanos, R., Rice, A., Rossetti, T., and Atkinson, K. (2007) Therapeutic applications of mesenchymal stromal cells. *Semin. Cell Dev. Biol.* **18**, 846–858.
6. Kidd, S., Spaeth, E., Klopp, A., Andreeff, M., Hall, B., and Marini, F.C. (2008) The (in) auspicious role of mesenchymal stromal cells in cancer: be it friend or foe. *Cytotherapy* **10**, 657–667.
7. Dwyer, R.M., Potter-Beirne, S.M., Harrington, K.A., Lowery, A.J., Hennessy, E., Murphy, J.M., Barry, F.P., O'Brien, T., and Kerin, M.J. (2007) Monocyte chemotactic protein-1 secreted by primary breast tumors stimulates migration of mesenchymal stem cells. *Clin. Cancer Res.* **13**, 5020–5027.
8. Lin, S.Y., Yang, J., Everett, A.D., Clevenger, C.V., Koneru, M., Mishra, P.J., Kamen, B., Banerjee, D., and Glod, J. (2008) The isolation of novel mesenchymal stromal cell chemotactic factors from the conditioned medium of tumor cells. *Exp. Cell Res.* **314**, 3107–3117.
9. Mishra, P.J., Humeniuk, R., Medina, D.J., Alexe, G., Mesirov, J.P., Ganesan, S., Glod, J.W., and Banerjee, D. (2008) Carcinoma-associated fibroblast-like differentiation of human mesenchymal stem cells. *Cancer Res.* **68**, 4331–4339.
10. Dittmer, A., Hohlfeld, K., Lutzkendorf, J., Muller, L.P., and Dittmer, J. (2009) Human mesenchymal stem cells induce E-cadherin degradation in breast carcinoma spheroids by activating ADAM10. *Cell. Mol. Life Sci.* **66**, 3053–3065.
11. Edwards, D.R., Handsley, M.M., and Pennington, C.J. (2008) The ADAM metalloproteinases. *Mol. Aspects Med.* **29**, 258–289.
12. Hundhausen, C., Misztela, D., Berkhout, T.A., Broadway, N., Saftig, P., Reiss, K., Hartmann, D., Fahrenholz, F., Postina, R., Matthews, V., Kallen, K.J., Rose-John, S., and Ludwig, A. (2003) The disintegrin-like metalloproteinase ADAM10 is involved in constitutive cleavage of CX3CL1 (fractalkine) and regulates CX3CL1-mediated cell-cell adhesion. *Blood* **102**, 1186–1195.
13. Murai, T., Miyauchi, T., Yanagida, T., and Sako, Y. (2006) Epidermal growth factor-regulated activation of Rac GTPase enhances CD44 cleavage by metalloproteinase disintegrin ADAM10. *Biochem. J.* **395**, 65–71.
14. Mochizuki, S. and Okada, Y. (2007) ADAMs in cancer cell proliferation and progression. *Cancer Sci.* **98**, 621–628.
15. Arima, T., Enokida, H., Kubo, H., Kagara, I., Matsuda, R., Toki, K., Nishimura, H., Chiyomaru, T., Tatarano, S., Idesako, T., Nishiyama, K., and Nakagawa, M. (2007) Nuclear translocation of ADAM-10 contributes to the pathogenesis and progression of human prostate cancer. *Cancer Sci.* **98**, 1720–1706.
16. Lee, S.B., Schramme, A., Doberstein, K., Dummer, R., Abdel-Bakky, M.S., Keller, S., Altevogt, P., Oh, S.T., Reichrath, J., Oxmann, D., Pfeilschifter, J., Mihic-Probst, D., and Gutwein, P. (2009) ADAM10 is upregulated in melanoma metastasis compared with primary melanoma. *J. Invest. Dermatol.* **130**, 763–773.
17. Sasser, A.K., Mundy, B.L., Smith, K.M., Studebaker, A.W., Axel, A.E., Haidet, A.M., Fernandez, S.A., and Hall, B.M. (2007) Human bone marrow stromal cells enhance breast cancer cell growth rates in a cell line-dependent manner when evaluated in 3D tumor environments. *Cancer Lett.* **254**, 255–264.
18. Yu, J.M., Jun, E.S., Bae, Y.C., and Jung, J.S. (2008) Mesenchymal stem cells derived from human adipose tissues favor tumor cell growth in vivo. *Stem Cells Dev.* **17**, 463–473.
19. Karnoub, A.E., Dash, A.B., Vo, A.P., Sullivan, A., Brooks, M.W., Bell, G.W., Richardson, A.L., Polyak, K., Tubo, R., and Weinberg, R.A. (2007) Mesenchymal stem cells within tumour stroma promote breast cancer metastasis. *Nature* **449**, 557–563.
20. Djouad, F., Bony, C., Apparailly, F., Louis-Plence, P., Jorgensen, C., and Noel, D. (2006) Earlier onset of syngeneic tumors in the presence of mesenchymal stem cells. *Transplantation* **82**, 1060–1066.
21. Djouad, F., Plence, P., Bony, C., Tropel, P., Apparailly, F., Sany, J., Noel, D., and Jorgensen, C. (2003) Immunosuppressive effect of mesenchymal stem cells favors tumor growth in allogeneic animals. *Blood* **102**, 3837–3844.
22. Parekkadan, B., van Poll, D., Suganuma, K., Carter, E.A., Berthiaume, F., Tilles, A.W., and Yarmush, M.L. (2007) Mesenchymal stem cell-derived molecules reverse fulminant hepatic failure. *PLoS One* **2**, e941.
23. Khakoo, A.Y., Pati, S., Anderson, S.A., Reid, W., Elshal, M.F., Rovira, I.I., Nguyen, A.T., Malide, D., Combs, C.A., Hall, G., Zhang, J., Raffeld, M., Rogers, T.B., Stetler-Stevenson, W., Frank, J.A., Reitz, M., and Finkel, T. (2006) Human mesenchymal stem cells exert potent antitumorigenic effects in a model of Kaposi's sarcoma. *J. Exp. Med.* **203**, 1235–1247.
24. Nakamura, K., Ito, Y., Kawano, Y., Kurozumi, K., Kobune, M., Tsuda, H., Bizen, A., Honmou, O., Niitsu, Y., and Hamada, H. (2004) Antitumor effect of genetically engineered mesenchymal stem cells in a rat glioma model. *Gene Ther.* **11**, 1155–1164.
25. Ohlsson, L.B., Varas, L., Kjellman, C., Edvardsen, K., and Lindvall, M. (2003) Mesenchymal progenitor cell-mediated inhibition of tumor growth in vivo and in vitro in gelatin matrix. *Exp. Mol. Pathol.* **75**, 248–255.
26. Qiao, L., Xu, Z., Zhao, T., Zhao, Z., Shi, M., Zhao, R.C., Ye, L., and Zhang, X. (2008) Suppression of tumorigenesis by human mesenchymal stem cells in a hepatoma model. *Cell Res.* **18**, 500–507.
27. Fierro, F.A., Sierralta, W.D., Epunan, M.J., and Minguell, J.J. (2004) Marrow-derived mesenchymal stem cells: role in epithelial tumor cell determination. *Clin. Exp. Metastasis* **21**, 313–319.
28. Hombauer, H. and Minguell, J.J. (2000) Selective interactions between epithelial tumour cells and bone marrow mesenchymal stem cells. *Br. J. Cancer* **82**, 1290–1296.

29. Dittmer, A., Schunke, D., and Dittmer, J. (2008) PTHrP promotes homotypic aggregation of breast cancer cells in three-dimensional cultures. *Cancer Lett.* **260**, 56–61.
30. Debnath, J. and Brugge, J.S. (2005) Modelling glandular epithelial cancers in three-dimensional cultures. *Nat. Rev. Cancer* **5**, 675–688.
31. Lee, J.M., Dedhar, S., Kalluri, R., and Thompson, E.W. (2006) The epithelial-mesenchymal transition: new insights in signaling, development, and disease. *J. Cell Biol.* **172**, 973–981.
32. Hanahan, D. and Weinberg, R.A. (2000) The hallmarks of cancer. *Cell* **100**, 57–70.

**This article should be cited as follows**:

Dittmer, J. (2010) Mesenchymal stem cells: "repair cells" that serve wounds and cancer? *TheScientificWorldJOURNAL* **10,** 1234–1238. DOI 10.1100/tsw.2010.119.
